# Supplementary figures and images for: Proline-based solution maintains cell viability and stemness of canine adipose-derived mesenchymal stem cells after hypothermic storage
Source: PLoS One. 2022 Mar 1;17(3):e0264773. doi: 10.1371/journal.pone.0264773 (PMC8887718; doi:10.1371/journal.pone.0264773)

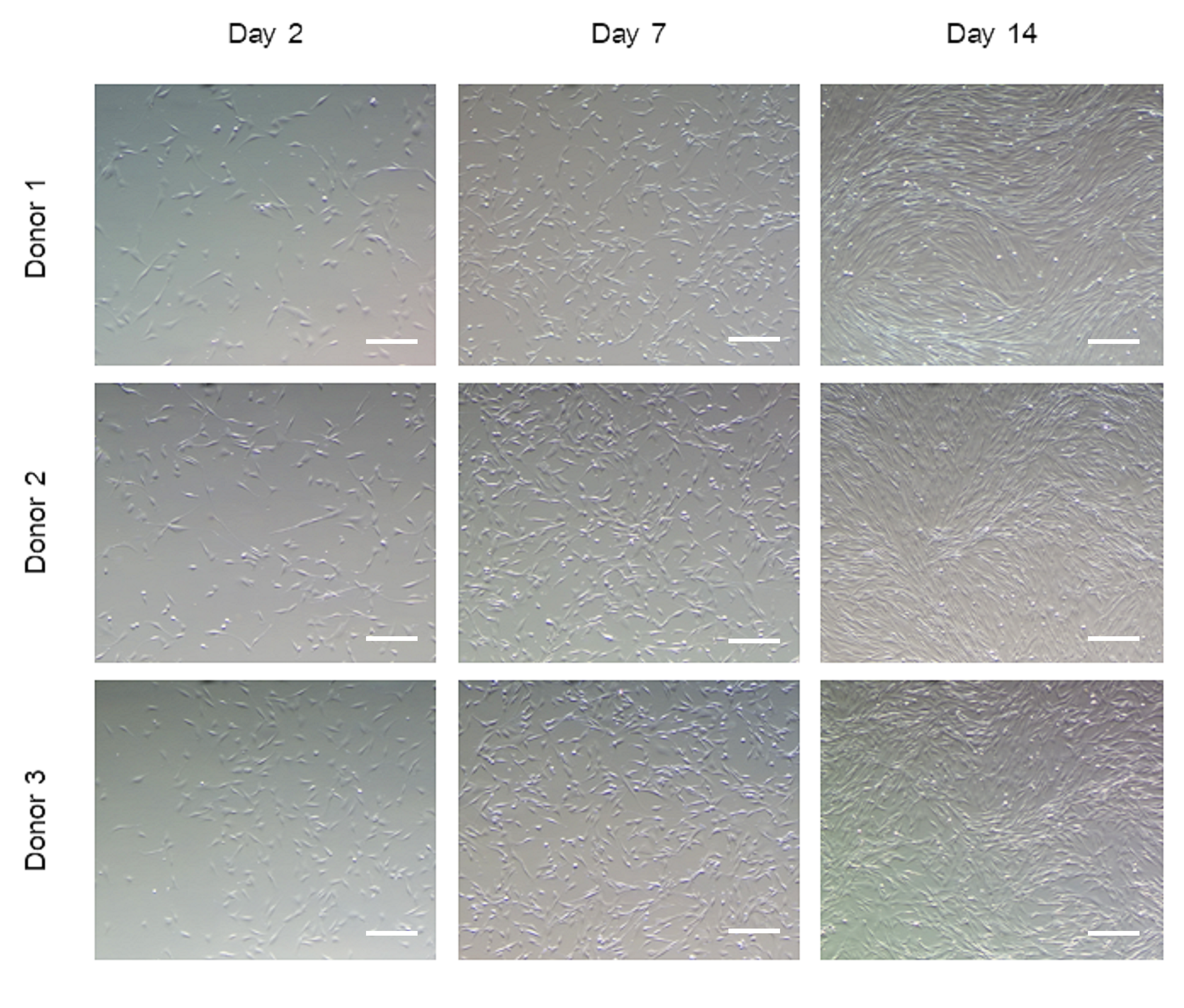

Supplement: S1 Fig — After 2 days of isolation, the adherent cells with different morphology, including fibroblast-like or epithelial-like shapes, were observed in the culture dishes. After 7 days, the cells had become more homogenous and those with a fibroblast-like shape had become dominant. The cells reached 80–90% confluence at 14 days of isolation. There was no notable difference in cell morphology among the three cell lines that had been isolated from different donors. All figures were performed at 10x magnification. Scale bars = 300 μm. (TIF) [file pone.0264773.s001.tif]

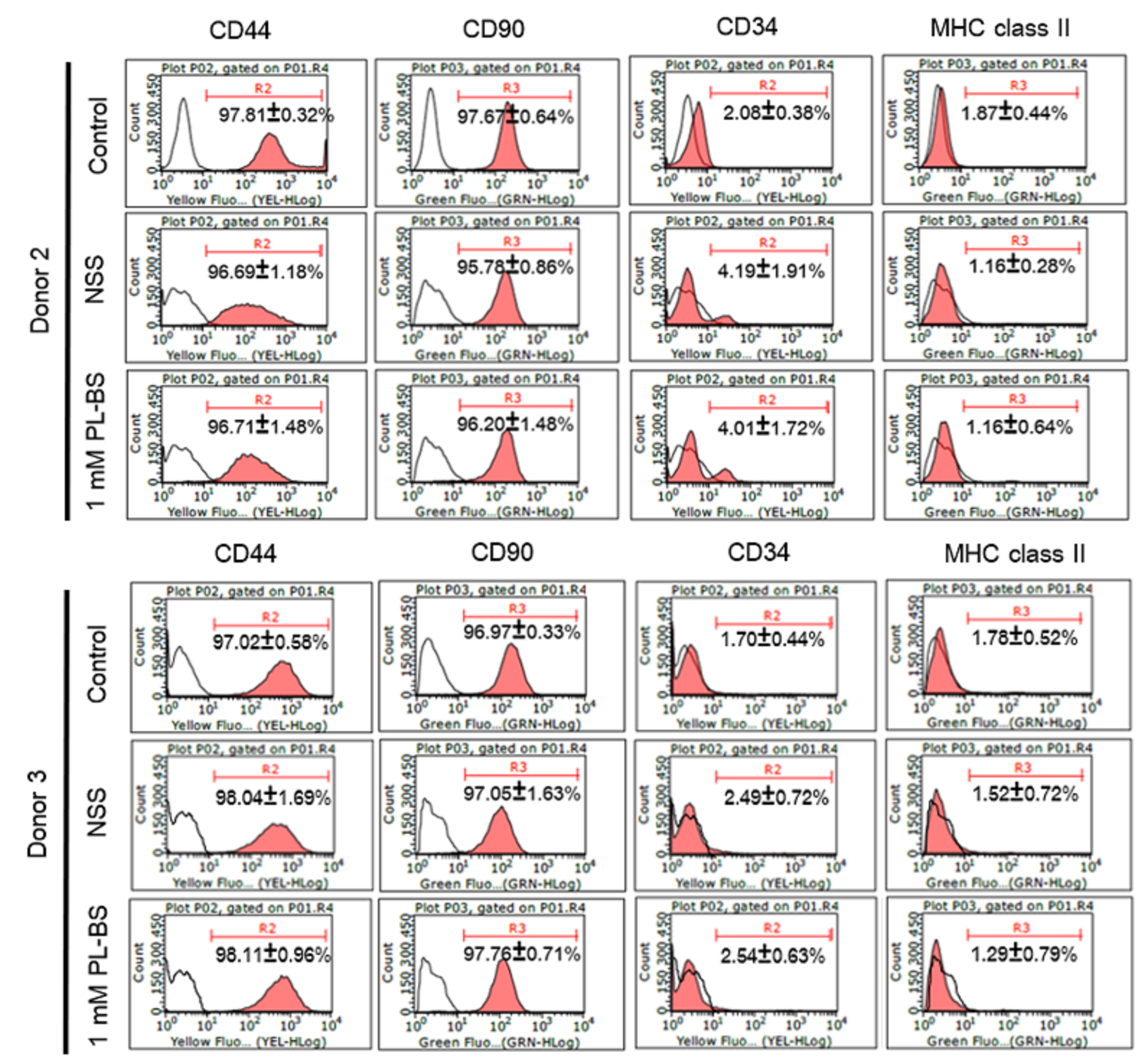

Supplement: S2 Fig — The stored cells in NSS and 1 mM PL-BS highly expressed both CD44 and CD90 but were deprived of both CD34 and MHC class II. These results were not different from those of the control group. (TIF) [file pone.0264773.s002.tif]

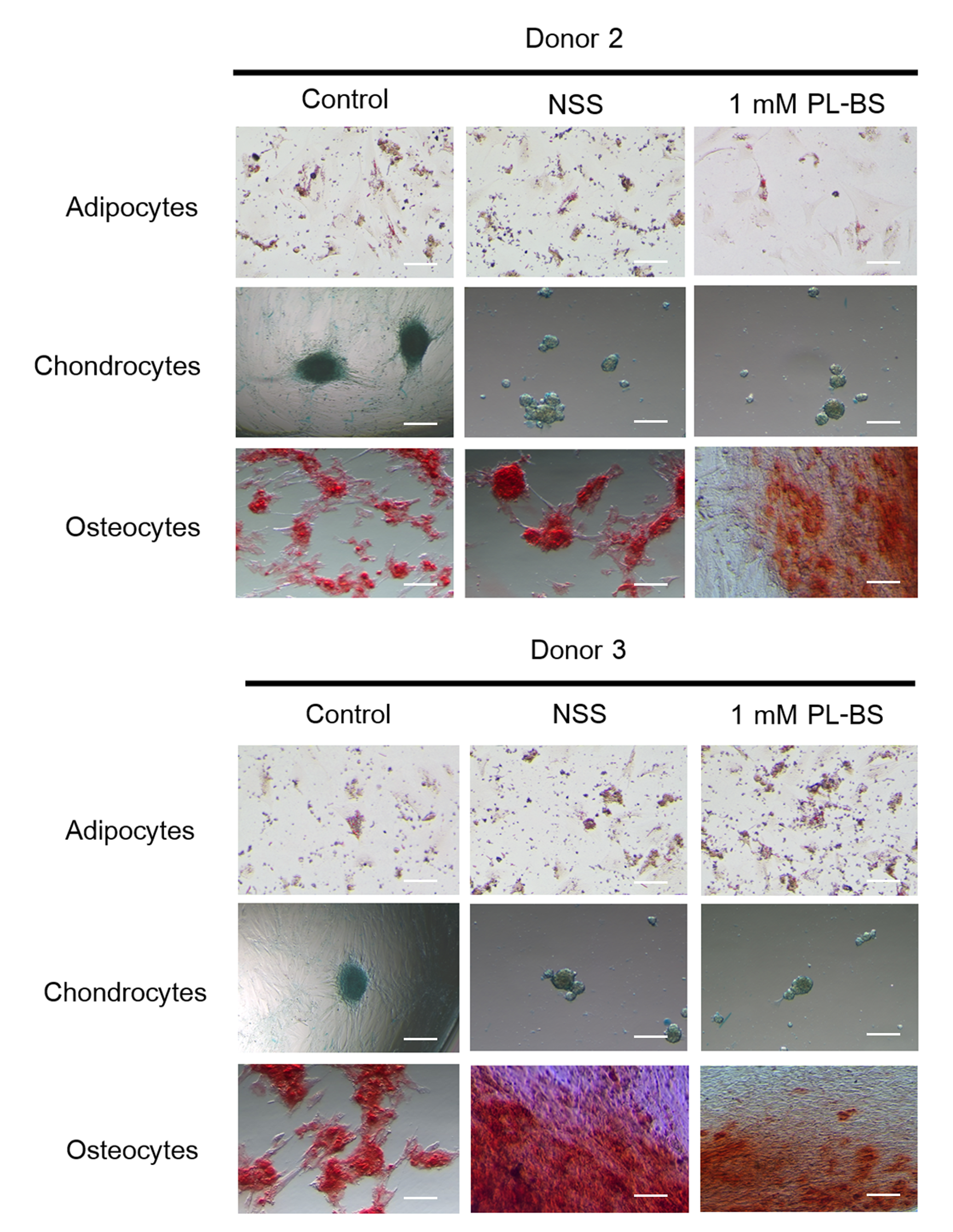

Supplement: S3 Fig — The post-stored cAD-MSCs exhibited differentiability into three lineages, including adipocytes, chondrocytes, and osteocytes. The fat droplets in adipocytes are stained red, indicating adipogenic differentiation potential. The blue color points out that chondrocytes are capable of producing proteoglycan in the extracellular matrix. The osteocytes with calcium accumulation are shown in red. Scale bars = 300 μm. (TIF) [file pone.0264773.s003.tif]

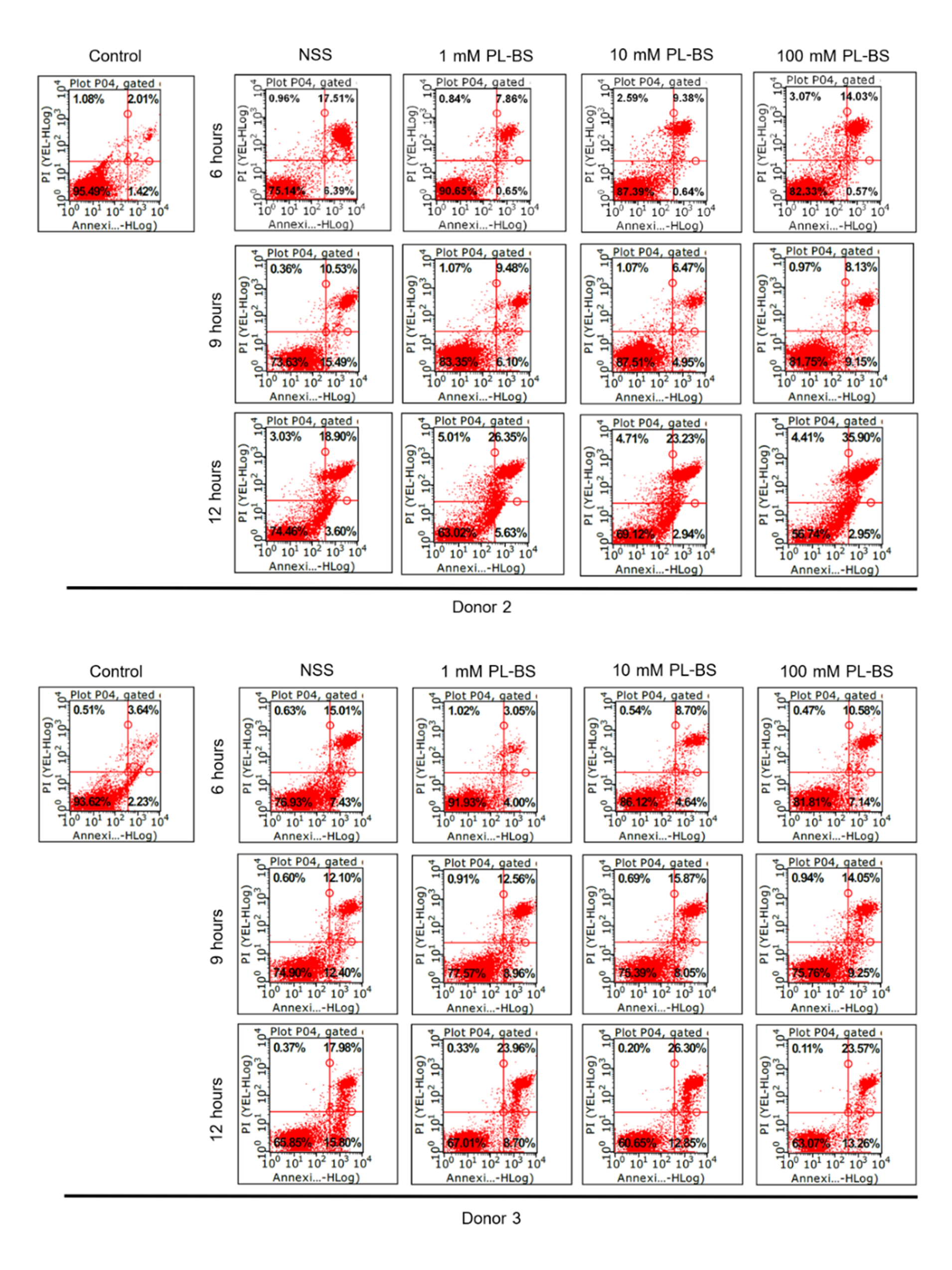

Supplement: S4 Fig — The cAD-MSCs from two other donors decreased the number of live cells in a time-dependent manner during hypothermic conditions. However, storage of cAD-MSCs in 1 mM PL-BS for 6 hours considerably improved cell viability by decreasing early apoptosis and necrosis in comparison with storage in other concentrations of both PL-BS and NSS. (TIF) [file pone.0264773.s004.tif]
