# Supplementary material for: Proline-based solution maintains cell viability and stemness of canine adipose-derived mesenchymal stem cells after hypothermic storage
Source: PLoS One. 2022 Mar 1;17(3):e0264773. doi: 10.1371/journal.pone.0264773 (PMC8887718; doi:10.1371/journal.pone.0264773)
Supplement: S1 Table — (DOCX) [file pone.0264773.s005.docx]

**S1** **Table. Sequence of primers used for evaluating of the expression of immunomodulatory genes.**

| **Gene** | **Primer sequence (5’-3’)** | **Amplicon size** | **Reference** |
| --- | --- | --- | --- |
| IDO | Forward - GCTGGGTCTGCCTCCTATTC  Backward - GCAGTCTCCACCAGGAAACC | 126 | [1] |
| HGF | Forward - GGCTACTGCTCCCAAATTCCA  Backward - CCACATTGAACATGTTAGTCCAGA | 123 | [1] |
| PGE-2 | Forward - CTGTCATCACCGGCCAAGT  Backward - CCTGGTCACTCCGGCAATA | 99 | [1] |
| IL-6 | Forward - AGGCTATCTTGCAGTCGCAG  Backward - TGCCCAGGCTACATTATCCG | 121 | This study |
| 18s | Forward - CCTGCGGCTTAATTTGACTC  Backward - CTGTCAATCCTGTCCGTGTC | 65 | [1] |

**Reference**

1. de Oliveira Pinheiro A, Lara VM, Souza AF, Casals JB, Bressan FF, Fantinato Neto P, et al. Characterization and Immunomodulation of Canine Amniotic Membrane Stem Cells. Stem Cells Cloning. 2020; 13: 43-55. doi: 10.2147/SCCAA.S237686.
