# Supplementary material for: Proline-based solution maintains cell viability and stemness of canine adipose-derived mesenchymal stem cells after hypothermic storage
Source: PLoS One. 2022 Mar 1;17(3):e0264773. doi: 10.1371/journal.pone.0264773 (PMC8887718; doi:10.1371/journal.pone.0264773)
Supplement: S2 Table — The gene expression results were quantified by normalization using the 2-ΔΔCT method and are shown as Log2 values of relative gene expression. (DOCX) [file pone.0264773.s006.docx]

**S2 Table. The effect of a hypothermic solution on the expression of immunomodulatory genes.**

| **Gene** | **Group** | **Donor 1** | **Donor 2** | **Donor 3** | **Mean** | **S.D.** |
| --- | --- | --- | --- | --- | --- | --- |
| **IDO** | **Control** | 0 | 0 | 0 | 0 | 0 |
|  | **NSS** | 4.9 | 3.98 | 3.59 | 4.16 | 0.67 |
|  | **1 mM PL-BS** | 5.68 | 4.35 | 4.20 | 4.74 | 0.81 |
| **IL-6** | **Control** | 0 | 0 | 0 | 0 | 0 |
|  | **NSS** | 3.24 | 3.14 | 2.98 | 3.12 | 0.13 |
|  | **1 mM PL-BS** | 2.99 | 2.81 | 2.58 | 2.79 | 0.21 |
| **HGF** | **Control** | 0 | 0 | 0 | 0 | 0 |
|  | **NSS** | 0.42 | 0.35 | 0.10 | 0.29 | 0.17 |
|  | **1 mM PL-BS** | 0.74 | 0.97 | 0.29 | 0.67 | 0.35 |
| **PGE-2** | **Control** | 0 | 0 | 0 | 0 | 0 |
|  | **NSS** | 2.98 | 3.52 | 3.16 | 3.22 | 0.27 |
|  | **1 mM PL-BS** | 1.55 | 0.16 | 0.78 | 0.83 | 0.70 |

The gene expression results were quantified by normalization using the 2^-ΔΔCT^ method and are shown as Log_2_ values of relative gene expression.
