# Supplementary material for: Proline-based solution maintains cell viability and stemness of canine adipose-derived mesenchymal stem cells after hypothermic storage
Source: PLoS One. 2022 Mar 1;17(3):e0264773. doi: 10.1371/journal.pone.0264773 (PMC8887718; doi:10.1371/journal.pone.0264773)
Supplement: Attachment — Submitted filename: Response to the Journal requirements.docx [file pone.0264773.s007.docx]

**Journal requirements:**

When submitting your revision, we need you to address these additional requirements.

1. Please ensure that your manuscript meets PLOS ONE&#39;s style requirements, including those for file naming. The PLOS ONE style templates can be found at

<https://journals.plos.org/plosone/s/file?id=wjVg/PLOSOne_formatting_sample_main_body.pdf>

and

<https://journals.plos.org/plosone/s/file?id=ba62/PLOSOne_formatting_sample_title_authors_affiliations.pdf>

Answer: We provided the manuscript according to PLOS ONE style requirements.

2. We suggest you thoroughly copyedit your manuscript for language usage, spelling,and grammar. If you do not know anyone who can help you do this, you may wish to consider employing a professional scientific editing service.

Whilst you may use any professional scientific editing service of your choice, PLOS has partnered with both American Journal Experts (AJE) and Editage to provide discounted services to PLOS authors. Both organizations have experience helping authors meet PLOS guidelines and can provide language editing, translation, manuscript formatting, and figure formatting to ensure your manuscript meets our submission guidelines. To take advantage of our partnership with AJE, visit the AJE website (<http://aje.com/go/plos>) for a 15% discount off AJE services. To take advantage of our partnership with Editage, visit the Editage website (www.editage.com) and enter referral code PLOSEDIT for a 15% discount off Editage services. If the PLOS editorial team finds any language issues in text that either AJE or Editage has edited, the service provider will re-edit the text for free.

Upon resubmission, please provide the following: The name of the colleague or the details of the professional service that edited your manuscript.

Answer: The language of our manuscript had been edited by the professional English-language editing staff at International College for Sustainability Studies of Srinakharinwirot University. The certificate of language editing is attached as a PDF.

```
A copy of your manuscript showing your changes by either highlighting them or using track changes (uploaded as a *supporting information* file)

Answer: Highlighting revised manuscript is provided and uploaded as a supporting  information file.
```

A clean copy of the edited manuscript (uploaded as the new *manuscript* file

Answer: A clean edited manuscript is provided and uploaded as the new manuscript file.

3. Thank you for stating the following financial disclosure:

&ldquo;This research was funded by the Faculty of Medicine, Srinakharinwirot

University (MED-GRAD-150; 511/2563 and MED-RES-200; 217/2563).&rdquo; Please state what role the funders took in the study. If the funders had no role, please state: &quot; The funders had no role in study design, data collection and analysis, decision to publish, or preparation of the manuscript.&quot;

If this statement is not correct you must amend it as needed.

Please include this amended Role of Funder statement in your cover letter; we will change the online submission form on your behalf.

Answer: Due to the funders had no role in the present study, therefore, we state “The funders had no role in study design, data collection and analysis, decision to publish, or preparation of the manuscript”.

4. Thank you for stating the following in the Acknowledgments Section of your

manuscript: &ldquo;This research was funded by the Faculty of Medicine, Srinakharinwirot University (MED-GRAD-150; 511/2563 and MED-RES-200; 217/2563).&rdquo; We note that you have provided information within the Acknowledgements Section. Please note that funding information should not appear in the Acknowledgments section or other areas of your manuscript. We will only publish funding information present in the Funding Statement section of the online submission form. Please remove any funding-related text from the manuscript and let us know how you would like to update your Funding Statement. Currently, your Funding Statement reads

as follows: This research was funded by the Faculty of Medicine, Srinakharinwirot University (MED-GRAD-150; 511/2563 and MED-RES-200; 217/2563).

Answer: The funding statement has been removed from the Acknowledgment section.

Please include your amended statements within your cover letter; we will change the online submission form on your behalf.

5. Please upload a new copy of

Figure 1A as the detail is not clear. Please follow the link for more information:

<https://blogs.plos.org/plos/2019/06/looking-good-tips-for-creating-your-plos-figures-graphics/>

Answer: The resolution of Figure 1A has been improved.

6. In your Data Availability statement, you have not specified where the minimal data set underlying the results described in your manuscript can be found. PLOS defines a study&#39;s minimal data set as the underlying data used to reach the conclusions drawn in the manuscript and any additional data required to replicate the reported study findings in their entirety. All PLOS journals require that the minimal data set be made fully available.

Answer: We uploaded the data set as the supporting information file (S2)

For more information about our data policy, please see <http://journals.plos.org/plosone/s/data-availability>.

Upon re-submitting your revised manuscript, please upload your studys minimal underlying data set as either Supporting Information files or to a stable, public repository and include the relevant URLs, DOIs, or accession numbers within your revised cover letter.

For a list of acceptable repositories, please see

<http://journals.plos.org/plosone/s/data-availability#loc-recommended-repositories>.

Any potentially identifying patient information must be fully anonymized.

Important: If there are ethical or legal restrictions to sharing your data publicly, please explain these restrictions in detail. Please see our guidelines for more information on what we consider unacceptable restrictions to publicly sharing data:

<http://journals.plos.org/plosone/s/data-availability#loc-unacceptable-data-access-restrictions>.

Note that it is not acceptable for the authors to be the sole named individuals responsible for ensuring data access.

We will update your Data Availability statement to reflect the information you provide in your cover letter.
